# Study on the failure law of surrounding rock in inclined coal seam with gob side entry

**DOI:** 10.1038/s41598-023-28238-3

**Published:** 2023-01-18

**Authors:** Hao Wu, Qingfeng Li, Chuanqu Zhu, Liao He

**Affiliations:** 1 College of Resources, Environment and Safety Engineering, Hunan University of Science and Technology, Xiangtan, 411201 China; 2 Institute of Mining Engineering, Hunan University of Science and Technology, Xiangtan, 411201 China

**Keywords:** Engineering, Civil engineering

## Abstract

Aiming at the problem of stability control of surrounding rock in the process of upward mining of inclined coal seam gob-side entry retaining, the gob-side entry retaining of 5449 working face in Beipingdong coal mine is taken as the research background. Based on the study of theoretical model of gob-side entry retaining, the law of overburden movement and surrounding rock deformation in the process of gob-side entry retaining is obtained through similar simulation experiment and numerical simulation analysis, and the optimal optimization scheme of supporting structure is put forward. The main research results are as follows: in the process of coal seam mining, the center of gravity of the overlying strata on the whole working face will shift to the side of the goaf, and the supporting structure composed of roadside filling body and bolt effectively inhibits the deformation of the roadway, in which the roadside filling body plays a key role; the top bolt bears greater stress than the side bolt in the process of mining, so the top bolt mainly maintains the stability of the roadway, and the side bolt plays the role of strengthening the side and preventing the extrusion. The width of the roadside filling body should be about 2.5 m, and the plastic failure range can be well controlled under this width.

## Introduction

The technology of retaining lanes along goaf has brought more favorable technical advantages and economic benefits to coal mining in China, and is a major breakthrough in the maintenance of lanes without coal pillars^1,2^. Chinese scholars have carried out a large number of studies on the application of this technology from different angles. He^3–5^ team has established a mechanical model of "surrounding rock structure—roadway side support body" under different roof positions, and based on this model, proposed the surrounding rock control technologies of roof cutting and pressure relief, such as concentrated presplitting blasting, constant resistance large deformation anchor cable, and dense single pillars beside the roadway, which laid a foundation for the successful implementation of roof cutting and pressure relief along the goaf. Zhu^6^ and others established the reliability analysis model of the support structure for gob side entry retaining in fully mechanized top coal caving according to the large deformation of surrounding rock and many random variables in the mechanical parameters of surrounding rock, and obtained the calculation formula for the reliability of the support structure. Zhang^7–10^ used "three high" bolts + anchor cable beam for initial support, anchor cable beam + grouting for advanced reinforcement, I-shaped steel beam + articulated roof beam for auxiliary support, paste material pumping and filling to construct the wall in the gob side entry retaining of high ground pressure soft rock roadway in a 1000 m deep well, effectively controlling the severe deformation of the side and roof of the gob side entry retaining in deep. Hua^11–13^ conducted an in-depth study on the deformation and failure mechanism of surrounding rock of gob side entry under the condition of deep well composite roof, and proposed the support countermeasures and technical schemes of "step by step strengthening at all stages" and "strengthening surface protection" for gob side entry under the condition of deep well composite roof, which have achieved good results in production. At the same time, domestic and foreign experts have also made fruitful achievements in the aspects of dynamic disaster^14,15^, roadway side support^16,17^, and roadway surrounding rock deformation control^18–20^ in the process of gob side entry retaining.

It can be seen that gob side entry retaining is still a safe and efficient mining technology in the future coal mining^21–23^, but there are still many technical problems in this technology under the conditions of complex geological conditions, high dynamic pressure in the deep and other conditions. Therefore, this paper takes the working face of gob side entry retaining for upward mining of an inclined coal seam in Beipingdong Coal Mine as the background, studies the failure law of surrounding rock in gob side entry retaining for upward mining of inclined coal seams, and optimizes the support structure based on the deformation and failure law of surrounding rock, provide ideas for safe mining of goaf side entry in similar inclined coal seams.

## Project overview

**Figure 1:**
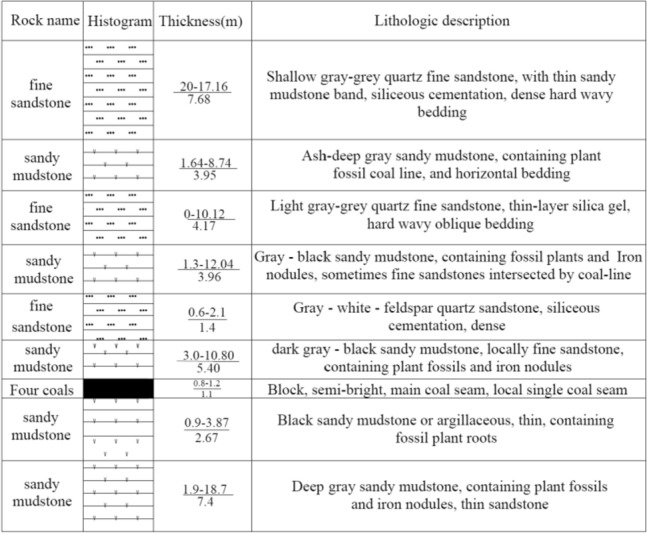
Comprehensive histogram of 5449 working face.

The 5449 working face of Beipingdong Coal Mine is located in the wusi mining area. The east side of the working face is close to the 5447 working face, and the west side is the non mining area; The ground is high mountains without buildings. The 5449 air tunnel mainly adopts four coal seams with good quality. The coal seam thickness is 1.0–1.4 m, the average coal thickness is 1.2 m, the coal seam dip angle is about 29°, the working face slope is large, the dip angle range is 12°–55°, the average is 25°. The direct top of Coal Seam 4 in the working face is dark gray black sandy mudstone, containing plant fossils and iron nodules, with a thickness of 0–10.8 m and an average of 5.4 m; The direct bottom of the working face is gray black sandy mudstone, containing plant root fossils, with a thickness of 0.9–4.24 m and an average of 1.73 m; The main roof of the working face is 4.17 m fine sandstone. The histogram of the top and bottom of the coal seam is shown in Fig. 1.

**Figure 2:**
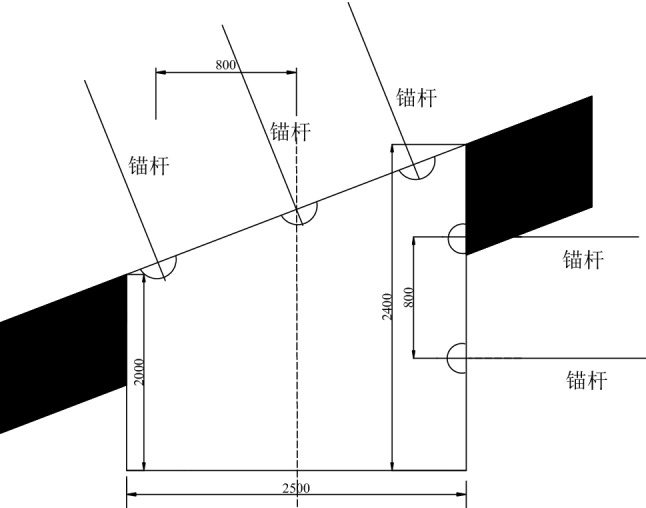
Bolt mesh section support of air roadway in 5449 working face.

The section of 5449 air roadway in Beipingdong Coal Mine is trapezoidal, the left side height of the roadway is 2000 mm, the right side height is 2400 mm, the roadway width is 2500 mm, and the net section area of the entire roadway is 4.4m^2^. The roadway section and anchor bolt layout are shown in Fig. 2.

## Similarity simulation of overburden movement and surrounding rock deformation

Through the similar simulation experiment, the dynamic evolution law of overburden collapse under the technical condition of gob side entry retaining in 5449 working face of Beipingdong Coal Mine, as well as the influence of working face recovery on the deformation of roadway surrounding rock are analyzed, revealing the role of bolt in restraining roadway deformation during mining, thus obtaining the bolt that plays a key role in roadway support, and further optimizing the roadway bolt support layout.

### Determination of simulation parameters

The experimental simulation platform device can accommodate the model size of 2000 mm × 500 cm × 1400 mm. Based on the size of the model experimental device and the geological production conditions on site, a physical similarity model (length × width × height = 1500 mm × 500 mm × 1200 mm) was laid along the inclination of the working face. The similarity ratio between each physical quantity should satisfy the following relationship: Geometric similarity ratio $C_{L} = 20:1$, the clear height and width of the model roadway are 100 mm and 125 mm respectively, the brass threaded rod is used to simulate the anchor bolt. According to the geometric similarity ratio, the length of each anchor bolt is 900 mm, and the row spacing between anchor bolts is 40 mm; density similarity ratio is $C_{\gamma } = 2: 1$. Stress similarity ratio $C_{\sigma } = 40:1$, the uniform load applied on the upper part of the model is 0.225 MPa through similar conversion.

### Establishment of similar model

**Figure 3:**
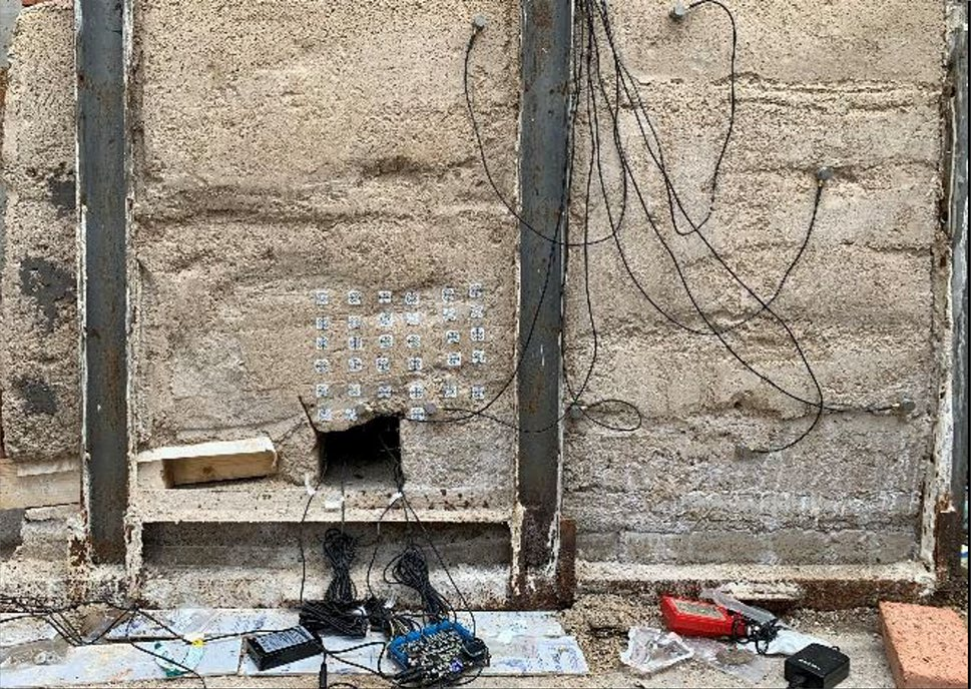
Overall view of the model.

In the model, river sand, lime, gypsum and water are uniformly mixed as similar materials according to the specified proportion for rock layers of different lithology. Timber strips are used to simulate coal seam mining. The proportion of materials used for similar material models is strictly in accordance with Table 1. The overall view of the model is shown in Fig. 3, and the location of each monitoring point is shown in Fig. 3. In order to prevent the boundary effect caused by the unsmooth four walls of the platform during the experiment, a plastic film is laid on the four walls of the test bench and applied with lubricating oil before laying the model. In the process of the experiment, the filling materials beside the roadway are paved as ordinary elastoplastic materials.

**Table 1:** Proportioning of Materials for Similar Material Models.

Name	Lithological characteristics	Model layer thickness/mm	River sediment mass/kg	Lime mass/kg	Gypsum mass/kg	Weight of water/kg	Number of layers
Overburde-n	Fine sandstone	200	149.25	25	25	25	4
	Sandy mudstone	200	156	9	22	19	4
	Fine sandstone	200	149.25	25	25	25	4
	Sandy mudstone	200	156	9	22	19	4
Main roof	Fine sandstone	60	44.775	5.625	5.625	5.625	2
Immediate Roof	Sandy mudstone	270	210.6	12.15	29.7	25.65	9
coal seam	Coal	75	81.75	8.25	3.375	9.375	3
Direct bottom	Sandy mudstone	85	66.3	3.825	9.35	8.075	2

### Experimental process and result analysis

**Figure 4:**
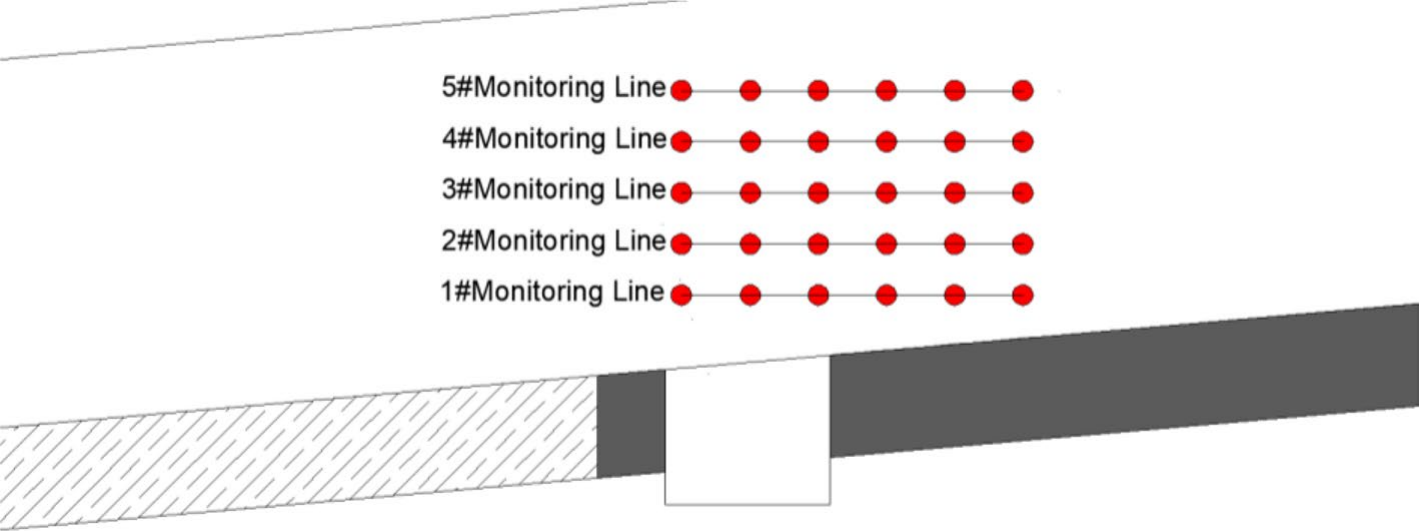
Layout of monitoring line.

During the experiment, five monitoring lines are arranged at 1 m, 2 m, 3 m, 4 m and 5 m above the coal seam, as shown in Fig. 4. The coordinates of monitoring points and initial coordinates of each mining are recorded, and the changes of overlying strata during mining are recorded.

#### Dynamic evolution characteristics of overburden collapse during mining

**Figure 5:**
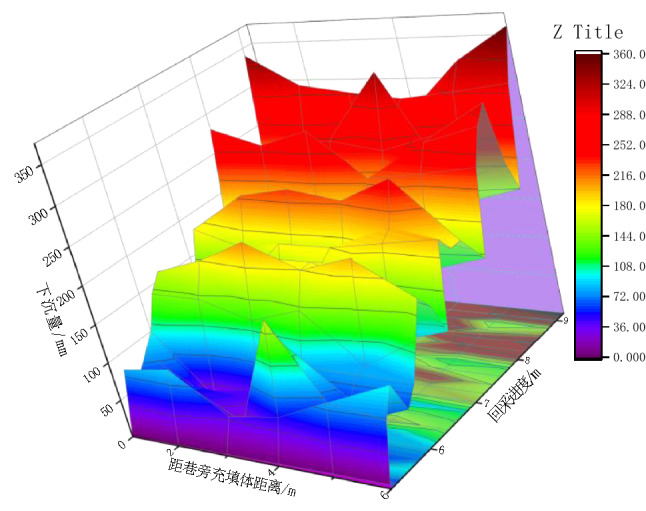
Change rule of vertical displacement of roof rock stratum above the working face during mining.

**Figure 6:**
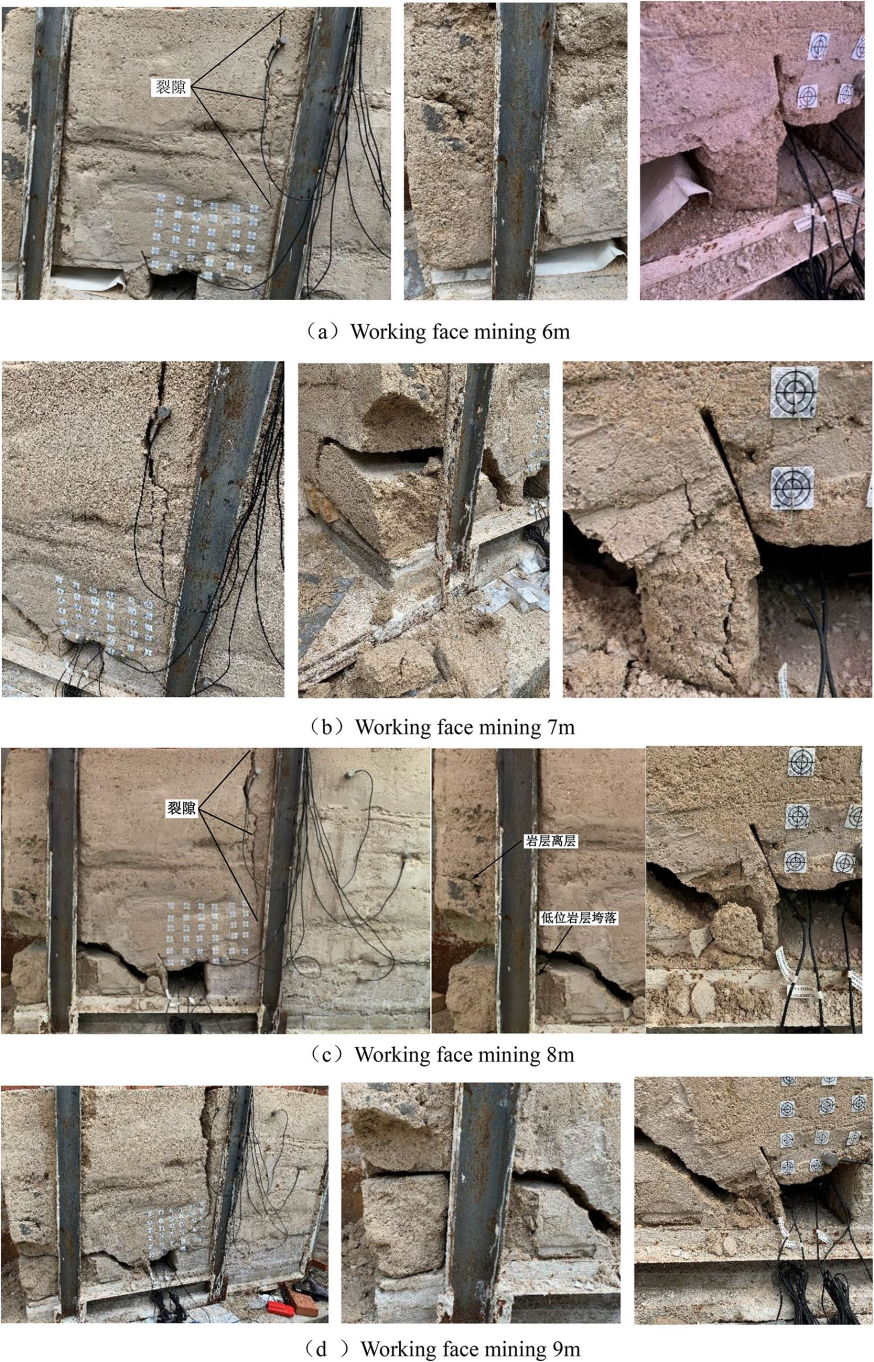
Overburden collapse process during mining.

The process of coal mining is often accompanied by the collapse of the overlying strata. As one of the important characteristic parameters, the displacement of the strata is closely related to the stability of the surrounding rock of the roadway. Therefore, the relationship between the five monitoring lines above the roadway and mining and the collapse process of the overlying strata are plotted, as shown in Figs. 5 and 6. Combined with Figs. 5 and 6, we can see the dynamic evolution law of overlying strata collapse under different mining progress.

(1) At the initial stage when the mining face reaches 6 m and 7 m, the roof subsidence starts to appear above the roadway, but the subsidence is small. The subsidence of the coal roof is greater than that of the roadway roof, and the maximum displacement reaches 182.1 mm at 2 m above the working face; due to the influence of mining, the strata above the side of the goaf are separated from each other. The separation range extends to 16 m above the goaf. The overlying strata have a large area of overhanging roof and are caving with mining. The filling body beside the roadway needs to bear the vertical stress of some overhanging roof strata. Therefore, a small part of the rock mass is squeezed off on the surface of the filling body beside the roadway.

(2) The collapse of all low level rock strata at the goaf side leads to a sharp increase in the subsidence of the overlying rock strata, which makes the stress concentrated at the goaf side, causing a new separation of high level rock strata. The collapsed rock is gradually compacted to support the high level rock strata, slightly slowing down the trend of bending and sinking of the high level rock strata. The maximum subsidence is located 5 m above the right side of the roadway, and the maximum subsidence reaches 359.6 mm; the crack at the upper right side of the roadway extends to the upper part of the roadway, and the entire overlying rock stratum breaks along the crack and settles toward the goaf side. As the only support structure, the roadway side filling body needs to bear the load of a larger area of suspended roof rock, so the roadway side filling body is severely squeezed and destroyed and loses its support capacity.

#### Evolution characteristics of bolt stress during mining

**Figure 7:**
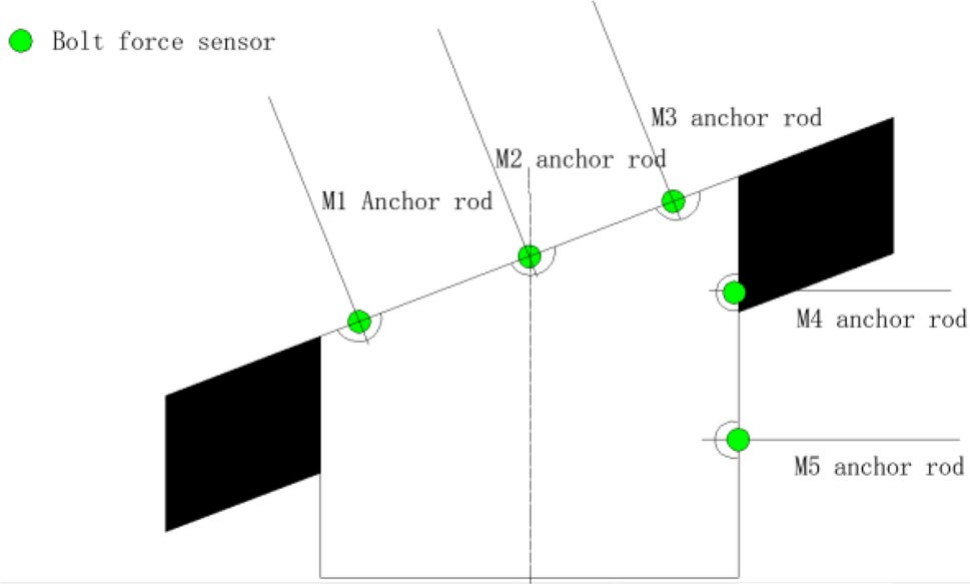
Distribution of anchor bolt and anchor bolt force sensor.

In the process of similar simulation experiment, the stress of anchor bolt is monitored in real time, and the distribution of anchor bolt and sensor is shown in Fig. 7.

**Figure 8:**
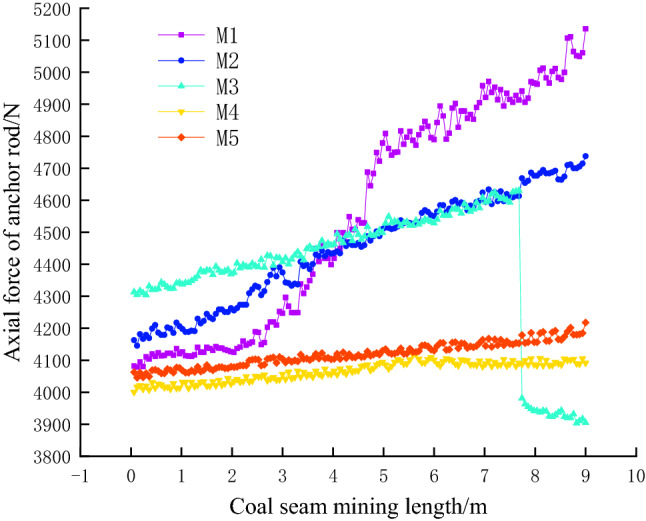
Stress variation of anchor rod.

During the experiment, a section of anchor bolt is set according to the layout of the anchor bolt in the air roadway of the 5449 working face of Beipingdong Coal Mine to monitor the axial stress of the anchor bolt during the mining process. The axial stress of the anchor bolt during the experiment is shown in Fig. 8.

As shown in Fig. 8, at the initial stage of mining, the stress amplitude of M1 anchor bolt starts to increase, and that of M2 anchor bolt also starts to increase, but is slightly smaller than that of M1, while that of M3, M4 and M5 anchor bolts is relatively gentle. When the mining is close to 8 m, M1 needs to bear the pressure of the roadway and the overlying strata in the goaf. Therefore, when the strata mining is completed and reaches equilibrium, the force on M1 bolt is the largest. When M3 bolt reaches the limit stress that it can withstand, it breaks and fails. Therefore, the force on the bolt suddenly drops sharply, and continues to decrease with the mining after falling to a certain value. The force growth rate of M4 and M5 anchor rods is stable and small, and the force of M5 anchor rod is always greater than that of M4 anchor rod in the whole process.

From this analysis, it can be seen that the roof bolts play a key role in restraining the roadway deformation during the mining process of the gob side roadway, especially the bolts on both sides of the roadway bear a large stress, and the side bolts mainly play a role in strengthening the stability of the side to prevent the occurrence of wall collapse.

## Optimization of support structure for gob side entry retaining

According to the law of rock stratum destruction in the similar simulation experiment, in the process of gob side entry retaining mining, the roadway side support is the main bearing structure, which plays a vital role in the stability of the roadway. In the experiment, the width of the roadway side fill was taken as a fixed value for the experiment. In this section, the roadway side support width was studied as a key variable, and the FLAC3D simulation software was used to simulate the distribution of plastic zones under different roadway side supports, the purpose is to simulate and determine the best filling body width beside the roadway.

### Establishment of numerical calculation model

**Figure 9:**
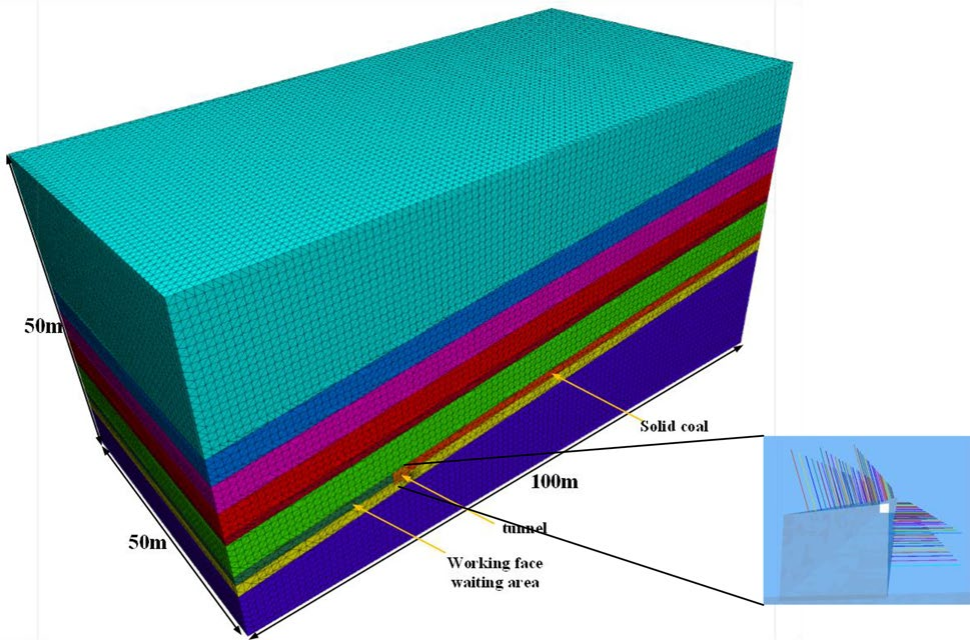
Schematic diagram of numerical calculation model.

Size of numerical simulation model: length × high × Width = 100 m × 50 m × 50 m. For the numerical model of 50 m, the average thickness of the coal seam is 1.2 m, the buried depth of the coal seam is 600 m, the unit weight of rock is 25KN/m3, the average density of rock stratum is 2700 kg/m3, and the initial vertical stress applied on the top of the model P = 9.0Mpa. The whole model is divided into 970,587 units and 174,743 nodes. The FLAC3D numerical simulation model is established as shown in Fig. 9. The FISH language embedded in FLAC3D is used to establish the support system of the numerical model according to the layout of the bolt in the original mine shaft roadway. The bolt is circularly driven into the model and a certain preload is applied. The model bolt support system is also shown in Fig. 9. The physical and mechanical properties of rock are shown in Table 2.

**Table 2:** Physical and mechanical properties of rock mass.

Coal seam and strata	Bulk modulus/GPa	Shear modulus /GPa	Tensile strength /Mpa	Cohesi-on /Mpa	Internal friction angle/°	Density/kg m^−3^
Sandy mudstone	8.85	5.93	0.1	2.71	32	2600
Four coals	3.8	4.21	4.38	4.21	32	1400
Fine sandstone	2.0	1.3	0.6	1.6	34	2700

In order to conform to the actual situation on site as much as possible, the Mohr Coulomb model is used in the calculation process. In the simulation process, it is assumed that the model is under hydrostatic pressure, that is, the set side pressure coefficient is 1. The displacement boundary conditions are applied to the front, rear, left, right and bottom of the model, while the stress boundary conditions are applied to the top. In this way, the lower boundary, front and rear boundaries, and left and right boundaries of the model are fixed. The whole model is under gravity acceleration, and a uniform load equal to the overburden weight is applied to the top.

### Influence of filling body width on distribution of plastic zone of roadway

**Figure 10:**
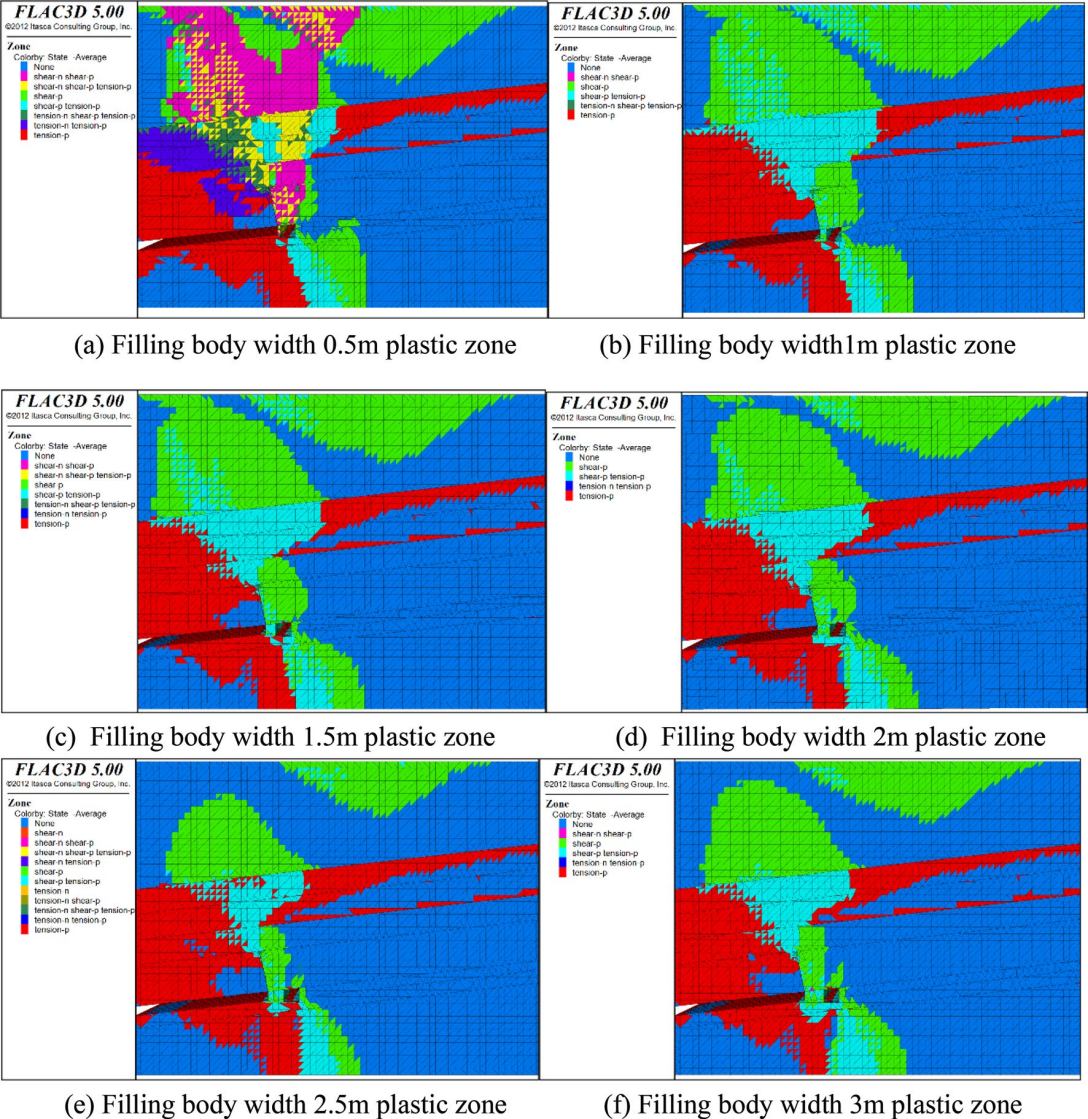
Distribution diagram of plastic distinction of different filling bodies.

The distribution characteristics of surrounding rock plastic zone is the key index to indicate the stability control of surrounding rock. The formation of surrounding rock plastic zone of roadway is the result of multiple factors. The shape of plastic zone is mainly affected by the stress state of surrounding rock. Therefore, this section studies the relationship between the width of the filling body and the distribution of the plastic zone, and studies the stability of the roadway from the perspective of the plastic zone. The cloud distribution diagram of the plastic division after the simulation calculation is shown in Fig. 10.

The comparative analysis of Fig. 10 shows that:

1. When the width of the filling body is 0.5 m and 1 m, the strength of the filling body is far from the requirement of supporting the roof due to its insufficient width, so the plastic zone is large at this time, the plastic failure zone is very developed and interconnected, the filling body beside the roadway has both tensile failure and shear failure, and the surrounding rock of the roadway is severely damaged.
2. When the filling body width is increased to 1.5 m and 2 m, the plastic area is much less than 0.5 m and 1 m. The plastic failure at the roadway roof begins to shift to the filling body roof and decreases. The roadway roof rock is mainly shear failure. When the filling body width is increased to 2.5 m and 3 m, the plastic zone area is significantly reduced, and the phenomenon that the plastic zones are interconnected can be better controlled.
3. It can be seen from Fig. 10e, f that when the width of the filling body increases to a certain value, the increase of the width does not make the plastic zone range decrease with the increase of the width, but tends to a stable state. Increasing the width of the filling body may lead to the increase of the plastic zone range in some areas. Therefore, the distribution range of the plastic zone and the width of the filling body are in a concave parabola relationship, considering the roadway stability, difficulty of roadway maintenance and economy, the best filling body width should be about 2.5 m.

## Conclusion

1. In the process of gob side entry retaining, the gravity center of the overlying strata of the whole working face will shift to the side of the goaf. The support structure composed of the roadway side filling body and anchor rod effectively inhibits the deformation of the roadway, and the roadway side filling body plays a key role.
2. In the bolt structure, the top bolt bears a large stress relative to the side bolt in the mining process, so the top bolt mainly maintains the stability of the roadway, and the side bolt strengthens the side and prevents the side from being squeezed.
3. The range of plastic zone of surrounding rock of roadway under different widths is simulated by numerical simulation, and the optimal filling body width should be about 2.5 m from the perspective of roadway stability, roadway maintenance difficulty and economy, so that the plastic failure range under the width can be well controlled.

## Data Availability

The data used to support the findings of this study are available from the corresponding author upon request.
